# Grey and White Matter Clinico-Anatomical Correlates of Disinhibition in Neurodegenerative Disease

**DOI:** 10.1371/journal.pone.0164122

**Published:** 2016-10-10

**Authors:** Alexander Frizell Santillo, Karl Lundblad, Markus Nilsson, Maria Landqvist Waldö, Danielle van Westen, Jimmy Lätt, Erik Blennow Nordström, Susanna Vestberg, Olof Lindberg, Christer Nilsson

**Affiliations:** 1 Clinical Memory Research Unit, Department of Clinical Sciences, Lund University, Lund, Sweden; 2 Lund University Bioimaging Centre (LBIC), Lund University, Lund, Sweden; 3 Geriatric Psychiatry Unit, Department of Clinical Sciences, Lund University, Lund, Sweden; 4 Center for Medical Imaging and Physiology, Skåne University Hospital, Lund, Sweden; 5 Department of Neurology and Rehabilitation Medicine, Skåne University Hospital, Lund, Sweden; 6 Department of Psychology, Lund University, Lund, Sweden; 7 Division of Clinical Geriatrics, Karolinska Institute, Stockholm, Sweden; University of Florida, UNITED STATES

## Abstract

Disinhibition is an important symptom in neurodegenerative diseases. However, the clinico-anatomical underpinnings remain controversial. We explored the anatomical correlates of disinhibition in neurodegenerative disease using the perspective of grey and white matter imaging. Disinhibition was assessed with a neuropsychological test and a caregiver information-based clinical rating scale in 21 patients with prefrontal syndromes due to behavioural variant frontotemporal dementia (*n* = 12) or progressive supranuclear palsy (*n* = 9), and healthy controls (*n* = 25). Cortical thickness was assessed using the Freesurfer software on 3T MRI data. The integrity of selected white matter tracts was determined by the fractional anisotropy (FA) from Diffusion Tensor Imaging. Disinhibition correlated with the cortical thickness of the right parahippocampal gyrus, right orbitofrontal cortex and right insula and the FA of the right uncinate fasciculus and right anterior cingulum. Notably, no relationship was seen with the thickness of ventromedial prefrontal cortex. Our results support an associative model of inhibitory control, distributed in a medial temporal lobe-insular-orbitofrontal network, connected by the intercommunicating white matter tracts. This reconciles some of the divergences among previous studies, but also questions the current conceptualisation of the “prefrontal” syndrome and the central role attributed to the ventromedial prefrontal cortex in inhibitory control.

## Introduction

Disinhibition is considered a cardinal feature in the prefrontal syndrome, [1–3]. More specifically, the classical clinico-anatomical correlate of disinhibition is the orbitofrontal cortex (OFC) [1–3]. This appears to apply also to neurodegenerative diseases, where disinhibition is associated with OFC atrophy [4–6]. However, a number of studies in these conditions imply considerably larger areas involved in behavioural inhibition including the temporal lobe, amygdala, hippocampus, caudate, insula and other parts of the prefrontal cortex (PFC) than the OFC [7–10]. Studies not finding any correlate to the OFC, have questioned the “OFC model” in its entirety [9]. The studies mentioned above have all correlated disinhibition to imaging of the grey matter, while similar approaches using white matter neuroimaging with Diffusion Tensor Imaging (DTI) are just emerging [11]. DTI is a method that gives the opportunity to describe the anatomy of white matter fibre bundles in vivo and to quantify changes in these tracts as a consequence of pathology [12]. In the context of correlative neuroanatomy, this enables a structural connectivity approach as a supplement to the cortical locationalist approach [13], which potentially can yield better models. In some conditions, the white matter imaging perspective has been able to literally bridge apparent discrepancies in assessments of cortical function. Psychopathy, for example, has been alternately associated with aberrations in the OFC and/or the right anterior temporal lobe, which may be reconciled by the findings of loss of altered diffusion properties of the white matter fibre bundle connecting these regions, i.e. the uncinate fasciculus [14].

In the current study we used a mixed cohort of patients with prefrontal syndromes with a diagnosis of either behavioural variant frontotemporal dementia (bvFTD) or progressive supranuclear palsy (PSP). These disorders were chosen since they have overlapping cognitive and behavioural deficits, with a variable extent of prefrontal syndrome severity [15–19], and are biologically similar with overlapping topography, neuropathology and genetics [20–22]. In bvFTD atrophy predominantly affects the prefrontal (including OFC), anterior temporal and insular cortex [23] with corresponding affliction of the basal ganglia [24], with the emergence of corresponding symptoms: apathy, disinhibition, dysphasia, loss of insight and executive deficits [25]. In PSP, the pathology always affects the brainstem and basal ganglia and to a variable extent the prefrontal cortex, resulting in a prefrontal-subcortical syndrome [6, 16, 22]. This is dominated by apathy but includes disinhibited behaviours, albeit being less frequent [15, 26]. As bvFTD/PSP represent a continuum of neurodegeneration of the prefrontal-subcortical systems corresponding to a varying degree of disinhibition, a mixed bvFTD/PSP cohort is favourable compared with the bvFTD and Alzheimer´s disease (AD) cohorts (two considerably more diverse disorders) when used in correlational studies of neurodegenerative disease [11, 27].

The primary aim of this study was to explore the neuroanatomical basis of disinhibition. To this purpose, measurements of cortical thickness and white matter integrity of fibre bundles leading to/from the OFC were correlated with results of a neuropsychological test of disinhibition and quantifications of behavioural disturbance using caregiver-based information. We hypothesised that by examining the correlation of behavioural measures to grey *and* white matter, we could provide an explanation for the conflicting results in previous grey matter only correlation studies. Also, direct comparisons of grey and white matter atrophy between the two prefrontal syndromes in our cohort (bvFTD and PSP) are rare and as such constituted a secondary aim of the study.

## Methods

### Participants

Participants were from the Lund Prospective Frontotemporal Dementia Study (LUPROFS), a longitudinal study of patients with any of the frontotemporal dementia spectrum disorders. Diagnostic workup included clinical examination, caregiver history with rating of behavioural disturbances and general disease severity, standardized neurological examination, MRI according to study protocol, and a comprehensive neuropsychological assessment. Selection criteria for the present study was a possible, probable or definite diagnosis of bvFTD or PSP according to FTDC [28] or NINDS criteria [29], respectively, completed MRI and availability of the Hayling neuropsychological test (described below) [30], which was part of an extended neuropsychological test protocol. Among the bvFTD patients (*n* = 12), 9 had probable and 3 definite bvFTD (genetic mutation carriers). Cerebrospinal fluid (CSF) was analysed for Alzheimer´s Disease (AD) core biomarkers (amyloid-ß 1–42, tau and p-tau) and a CSF biomarker profile indicative of AD [31], was used as an exclusion criterion [28]. Of the PSP patients (*n* = 9), 1 had possible and 8 probable PSP [29]. All patients except one were right-handed. Healthy controls (*n* = 25) underwent clinical interview and examination, including neuropsychological testing and MRI. Demographic and clinical data on the subjects are presented in Table 1.

**Table 1:** Demographic and clinical data of subjects.

	HC	bvFTD	PSP	Across	bvFTD vs. HC	PSP vs. HC	bvFTD vs. PSP
Number	25	12	9	-	-	-	-
Sex	13M 12F	5M 7F	3M 6F	n.s.	-	-	-
Age	68 (35–82)	71.5 (57–77)	67 (58–76)	n.s.	-	-	-
Mini Mental Status Examination	30 (29–30)	26 (21–30)	28 (24–30)	*p* < 0.001	*p* < 0.001	*p* = 0.004	n.s.
Education	14 (8–14)	9 (7–14)	11 (7–16)	n.s.	-	-	-
Frontotemporal Lobar Degeneration modified Clinical Dementia Rating scale	n.a.	8.5 (2–15)	4 (0.5–10.5)	-	-	-	n.s.
Duration	n.a.	2.5 (1–12)	4 (2–6)	-	-	-	n.s.
Frontal Behavioural Inventory^1-10^	n.a.	14 (6–21)	7 (2–17)	-	-	-	n.s.
Frontal Behavioural Inventory^12-22^	n.a.	9 (1–20)	5 (1–7)	-	-	-	*p* = 0.012
The Hayling test	4.5 (0–20)	23 (5–72)	7 (1–56)	*p* = 0.001	*p* < 0.001	n.s.	n.s.

Data are median values, with range. Age: age at examination in years, bvFTD: behavioural variant frontotemporal dementia, Duration: disease duration (in years), Education: education (in years), Frontal Behavioural Inventory^1-10^ and Frontal Behavioural Inventory^12-22^: sum of items 1–10 and 12–22, respectively, The Hayling test: total error score on the Hayling test, HC: healthy controls, n.a: not applicable, n.s.: not significant, PSP: progressive supranuclear palsy.

### Ethics statement

All participating subjects were informed of the study content in both oral and written form. Informed consent was taken in written form from the study subjects. If there was a doubt that a study subject had a compromised capacity to consent, informed consent was taken from the study subject and the spouse, or the spouse only. Importantly, regardless from whom informed consent was taken, the study subject always retained the right to decline or interrupt participation at any time. No formal assessment of the patients´ capacity to consent was performed. This procedure for informed consent, as well as all other aspects of the study, are according to the Helsinki declaration and were approved by the Regional Ethical Review Board, Lund, Sweden (Permit number 617/2008).

### Neuropsychological measure of disinhibition

The Hayling Sentence Completion Test, often referred to as just the Hayling test, is designed to measure response initiation and inhibition of response by a sentence completion task [30]. In the first part of the test, subjects are asked to complete a sentence with an appropriate word. For instance, the examiner reads aloud *“The rich child attended a private…”*, where *“school”* is a correct response. In the second part of the test, subjects are asked to complete a sentence with a word that is not at all appropriate, thus requiring inhibiting of an automatic response. An example from this part of the test is: *“London is a very lively…”*, where *“city”* is an incorrect answer, but *“banana”*, *“pet”*, or *“shoe”* are examples of correct answers. Errors in the second part are recorded and form a total error score, in addition to time to complete for the first and the second parts, which together are transformed into an overall score. The construct validity of the Hayling test has been examined in a limited number of studies. In comparison with other tests of executive functions, the Hayling test has shown positive correlations with the Six Elements test and subsequent time in the Tower of London test in psychiatric patients with clinical difficulties of inhibition [32, 33]. In healthy controls, there is a positive correlation between the Hayling test and the Stroop test regarding initiation whereas no association was found regarding the inhibitory components [34, 35], indicating that these tests measure a different dimension of inhibitory capacity. The Hayling test has been used previously for patients with bvFTD [11, 36], PSP [37] and appears to be more discriminative in bvFTD vs. AD compared with the more commonly used Stroop test [38]. The latter findings also support that the Hayling test does indeed reflect a different aspect of inhibition than the Stroop test. Patients with bvFTD generally show worse performance than controls on the Hayling test overall scaled score, second part scaled score, and the total error score (i.e. cumulative errors from the second part of the test) [11, 36]. For the current study the total error score was chosen, since this appears to be the most specific for bvFTD [11, 36].

### Behavioural and clinical severity rating

As a measure of behavioural inhibition in everyday life we used the Frontal Behavioural Inventory (FBI) [39]. The FBI is a rating scale based on a caregiver interview, administered by a health professional, with 24 items designed to capture core symptoms of bvFTD. It is widely used in studies on dementia and is more specific for “prefrontal” disturbances than more general neurobehavioural rating scales such as the Neuropsychiatric Inventory (NPI) [40]. The severity of single items is scored on a scale from 0 to 3 (not present, mild/occasional, moderate and severe/most of the time). The FBI is divided into two parts, where FBI items 1–10 represent negative symptoms such as apathy, emotional flatness, and personal neglect, and FBI item 12–22 represent positive symptoms with diminished inhibition such as perseverations, excessive jocularity, poor judgement, inappropriateness, impulsivity and hyperorality. For the current study, we used the sum of items 1–10 (FBI^1-10^) and the sum of items 12–22 (FBI^12-22^). For rating general disease severity we employed the sum of boxes in the Frontotemporal Lobar Degeneration modified Clinical Dementia Rating (FTLD-CDR) [41].

### MRI acquisition

MRI was performed using a Philips Achieva 3T scanner equipped with an eight-channel head coil. DTI data were acquired using a single-shot spin echo sequence with EPI using 48 diffusion encoding directions, a diffusion-weighting factor (b) of 800 s/mm^2^, voxel size 2x2x2 mm^3^, TR 7881 ms, and TE 90 ms. Motion and eddy current correction of the data was performed with ElastiX [42], by affine registration of the diffusion-weighted images to the first non-diffusion weighted image in the protocol [43]. The DTI protocol was followed by a T_1_-weighted 3D volumetric sequence with TR 8.3 ms, TE 3.84 ms, FOV 256x256x175 and a voxel size of 1x1x1 mm^3^.

### Cortical thickness analysis

Modeling and volumetric estimations of cortical brain regions were performed on structural T1 images using the Freesufer image analysis package version 5.3 (http://surfer.nmr.mgh.harvard.edu/). This automated tool performs imaging intensity normalization, removal of non-brain tissues, segmentation of cortical and subcortical brain regions into white and grey matter, spherical surface-based intersubject registration, which is based on the cortical surface curvature (sulci and gyri), and, finally, an automated parcellation of the cortical surface. Quality control of the Freesurfer output was done by visual inspection. When errors were noted the first approach was to edit these errors and, if errors remained after rerun of the edited output, the images were discarded from further analysis. The Query Design Estimate Contrast (QDEC) tool was used to do a general linear model (GLM) analysis at each vertex of the cortical surface. The dependent variable was cortical thickness. In the group comparisons, no nuisance variables were entered in to the model as groups were balanced with regards to gender and age. The results of the GLM analysis were corrected for multiple comparisons at the cluster level using the Monte Carlo method for p-cluster at *p* < 0.01 (z-vertex 2.0). In the correlation analysis between cortical thickness and clinical and neuropsychological data, age was entered as a nuisance variable and corrections for multiple comparisons were at the *p* < 0.01 (z-vertex 1.3) level.

### Tractography and postprocessing

**Fig 1:**
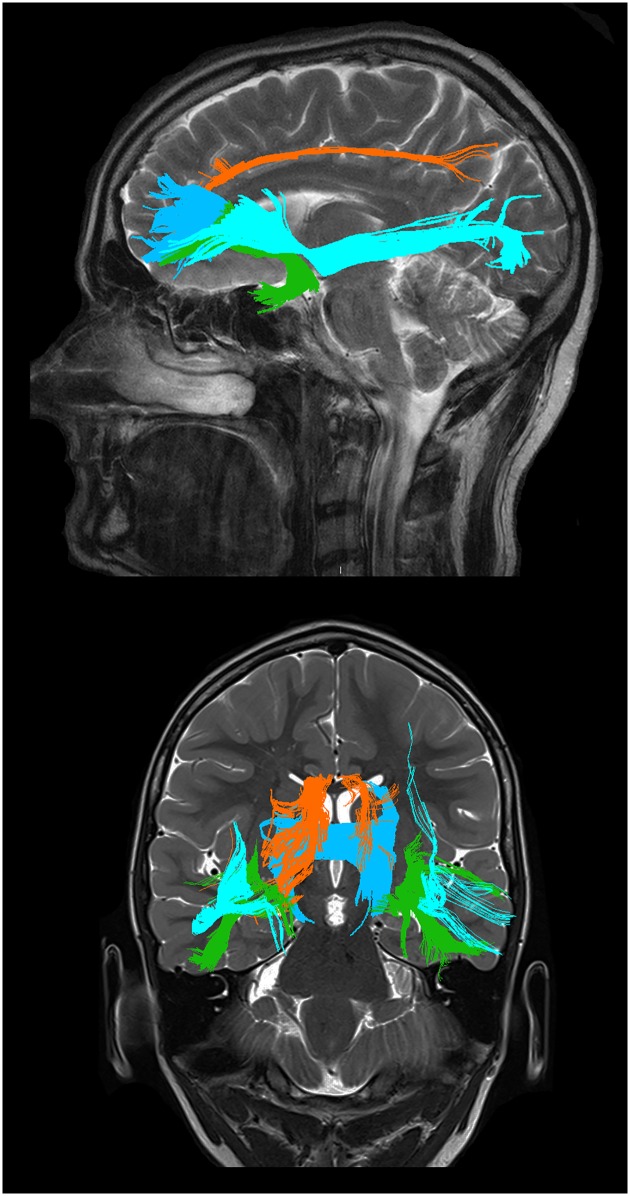
Graphical representations of the tracts studied. Sagittal (upper) and coronal (lower) view. The uncinate fasciculus (green), the anterior cingulum (orange), the inferior frontooccipital fasciculus (light blue) and the forceps minor (dark blue). Tracts are from TrackVis, overlaid on high resolution images for illustrative purposes.

Deterministic tractography was performed with an FA threshold of 0.2, an angular threshold of 45 degrees and no length threshold using the diffusion toolkit and TrackVis. ROIs manually drawn on FA maps were employed to dissect four pathways: the inferior frontooccipital fasciculus (IFOF), uncinate fasciculus (UF), anterior cingulum (aCi) and the forceps minor (Fig 1). For the IFOF, UF and aCi ROIs, methods described by Catani and co-workers were used [44]. Briefly, these consists of placing a ROI in the extreme capsule, which is linked with an occipital ROI to dissect the IFOF and linked with a ROI in the anterior temporal lobe to dissect the UF, respectively. For the cingulum, a single ROI covers the whole tract from its appearance ventral to the genu of the corpus callosum to its temporal terminations. We modified this approach to only include the anterior part of the cingulum, ending the ROI dorsally at the first slice where the corpus callosum appears continuous on axial slices. For the forceps minor we used two parasagittal ROIs over the anterior corpus callosum with a fixed dorsal extension, a procedure that will include the rostrum of the corpus callosum together with the ventral half of the genu, or area 1 and ventral half area 2 according to Witelson [45]. In all tractography procedures (except for the corpus callosum) a midsagittal ROI was placed to exclude commissural fibres. When apparent artefacts were generated, an additional exclusion ROI was drawn to exclude these. An attempt was made to dissect the anterior commissure but this resulted in low reliability. We also attempted an isolated subgenual parcellation of the cingulum. However, this resulted in a high proportion of either missing tracts or tracts composed of very few streamlines. All image analysis was done blinded for diagnosis. Intra- and inter-rater reliability (authors KL vs. KL and AFS vs. KL) for the tracking procedures were assessed on five patients and five controls using the volume of the tract in millilitres as the quantitative measure. In DTI there is a choice between different parameters, which appear to reflect different biological phenomena [46]. Since our primary questions were relationships between symptoms and integrity of different tracts, we chose the fractional anisotropy (FA) as our primary parameter. To corroborate our findings we used mean diffusivity (MD), which is closely related to axial and radial diffusivity (MD = [axial+radial diffusivity]/2), and less sensitive for partial volume effects in some circumstances [47], as a secondary parameter.

### Statistical analysis

Demographic, cognitive, behavioural and DTI parameters of tracts were compared across groups by the use of the Kruskal-Wallis test. Differences between group pairs were assessed with the Mann-Whitney U test. Distribution of gender was assessed by the Fisher´s Exact test. To examine the relationships between neuropsychological test results, behavioural data and DTI variables of tracts, we applied linear regression models adjusted with age. Model assumptions were checked and the logarithm of the dependent variable was used if needed to achieve normality and/or other model assumptions. The reliability of the tracking procedures was calculated using the intraclass correlations coefficient (ICC), single measures, and absolute agreement. Based on the effect sizes in previous group comparisons and correlative studies [48–50], the power of the current study (*n* = 21) is sufficient. The statistical analyses were performed in SPSS Statistics 21 for Mac (IBM Corporation, Somers, NY, USA). *p* values less than 0.05 were considered as statistically significant.

## Results

### Subject characteristics

Clinical and demographic characteristics of the patients and healthy controls are presented in Table 1. There were no statistically significant differences in age, education or sex distribution between the three groups. Also, there were no statistically significant differences in symptom duration or global cognitive level as measured with the FTLD-CDR or Mini Mental State Examination (MMSE) between patients with bvFTD and PSP. Patients with bvFTD had higher total FBI and FBI^12-22^ symptom score compared with PSP, but not FBI^1-10^ symptom scores, although the latter was borderline statistically significant (*p* = 0.054). The Hayling total error score showed a robust difference between bvFTD patients and controls, while PSP patients had values closer to controls. Although median total error score was higher in the bvFTD than the PSP group, this did not reach statistical significance (*p* = 0.055). All behavioural and neuropsychological measures (i.e. FTLD-CDR, FBI, the latter including subscores, and Hayling) showed considerable overlap in range, supporting the use of a combined bvFTD and PSP cohort.

### Correlations with the Hayling test

**Fig 2:**
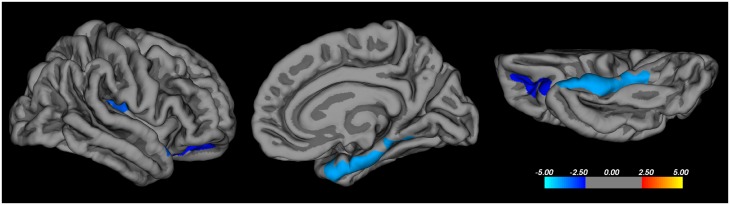
Cortical thickness and Hayling error score correlations. Correlations between cortical thickness and total error score on the Hayling test in patients with behavioural variant frontotemporal dementia and progressive supranuclear palsy using Freesurfer. Correction for multiple comparisons was made using the Monte Carlo method at the cluster level, at *p* < 0.01 (z-vertex 1.3). Age was entered as a nuisance variable. Coloured areas represent significant negative correlations, with the scale bar representing *p* on a logarithmic scale. Only the right hemisphere is shown, from lateral, medial and inferior views. No regions with significant correlation with cortical thickness were detected in the left hemisphere.

**Fig 3:**
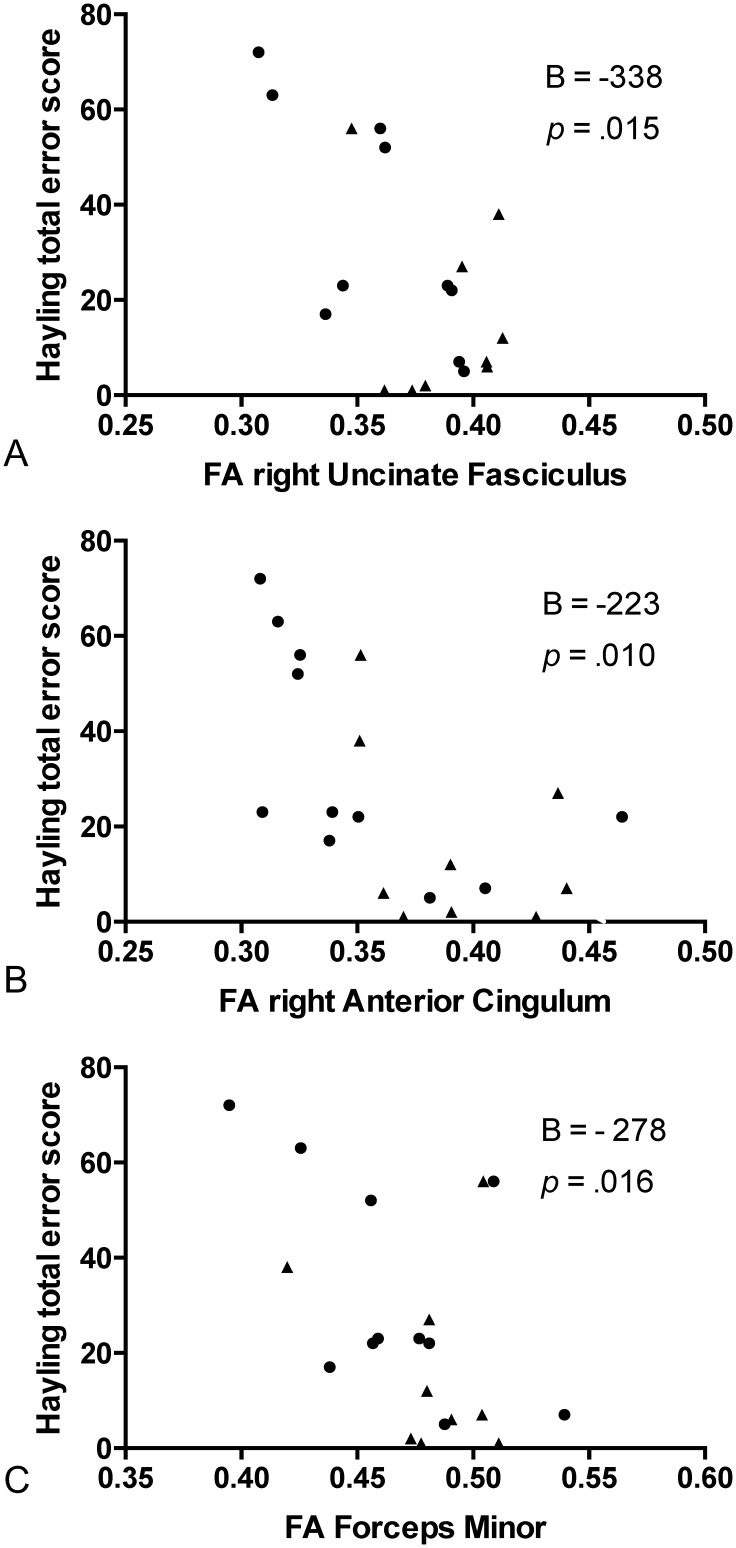
Fractional anisotropy and Hayling error score correlations. Scatter plots of total error score on the Hayling test and fractional anisotropy (FA) of tracts in patients with behavioural variant frontotemporal dementia (dots) and progressive supranuclear palsy (triangles), for A: the right uncinate fasciculus, B: the right anterior cingulum, and C: the forceps minor. B (adjusted) and *p* are derived from the linear regression model, with age as covariate.

In the combined patient cohort (bvFTD and PSP) there was a significant correlation between the Hayling total error score with cortical thickness in the following regions, confined to the right hemisphere: the OFC (excluding the ventromedial prefrontal cortex- vmPFC), the parahippocampal gyrus and the posterior insula (Fig 2). For white matter tracts, the linear regression model with Hayling total error score as the dependent and FA of the tract as the independent variable, was significant for the right UF (adjusted R^2^ = 0.55, unstandardized B = -338 [95% CI -601, -75], *p* = 0.015), right aCi (adjusted R^2^ = 0.56, B = -223 [95% CI -388, -60], *p* = 0.010), and forceps minor (adjusted R^2^ = 0.54, B = -278 [95% CI -496, -59], *p* = 0.016) (Fig 3), but not for any other tract. Age was a significant covariate in all models. All significant models were re-run with MD instead of FA as the DTI parameter of the tract, with the same models and parameters reaching statistical significance. The models were also re-run with gender as a covariate, which did not contribute significantly to the models.

### Correlations with the Frontal Behavioural Inventory

**Fig 4:**
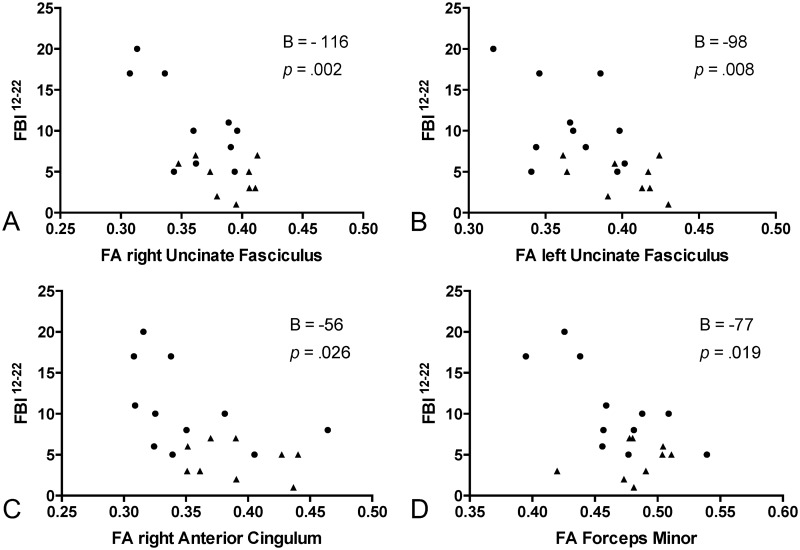
Frontal Behavioural Inventory and FA of tracts. Scatter plots of Frontal Behavioural Inventory composite score of items 12–22 (FBI^12-22^) and fractional anisotropy (FA) of tracts in patients with behavioural variant frontotemporal dementia (bvFTD) (dots) and progressive supranuclear palsy (PSP) (triangles), for A: the right uncinate fasciculus, B: the left uncinate fasciculus, C: the anterior cingulum and D: the forceps minor. B (adjusted) and *p* are derived from the linear regression model, with age as covariate.

In the linear regression model with the FBI^12-22^ of the combined patient cohort as the dependent variable, a statistically highly significant effect was seen for the right UF (adjusted R^2^ = 0.44, unstandardized B = -116 [95% CI -182, -50], *p* = 0.002), left UF (adjusted R^2^ = 0.319, B = -98 [95% CI -167, -29], *p* = 0.008), the right aCi (adjusted R^2^ = 0.23, B = -56 [95% CI -105, -7.6], *p* = 0.026) and forceps minor (adjusted R^2^ = 0.26, B = -77, [95% CI -139, -14], *p* = 0.019) (Fig 4). Age was not a significant covariate in the models. All significant models were re-run with MD instead of FA as the DTI parameter of the tract and showed the same results except for the right aCi, in which the MD model was not significant. The models were also rerun with gender as an additional covariate, which did not contribute significantly to the models. To examine to what extent the correlations were specific for symptoms of the FBI^12-22^, we computed the same analyses with FBI^1-10^ as the dependent variable. These analyses showed no significant effect of the DTI variables on FBI^1-10^ scores, but in several cases a significant effect of age. In the analysis of correlations between FBI scores and cortical thickness no significant correlation was seen with FBI^12-22^ nor FBI^1-10^ in any region.

### Group comparisons of cortical thickness

**Fig 5:**
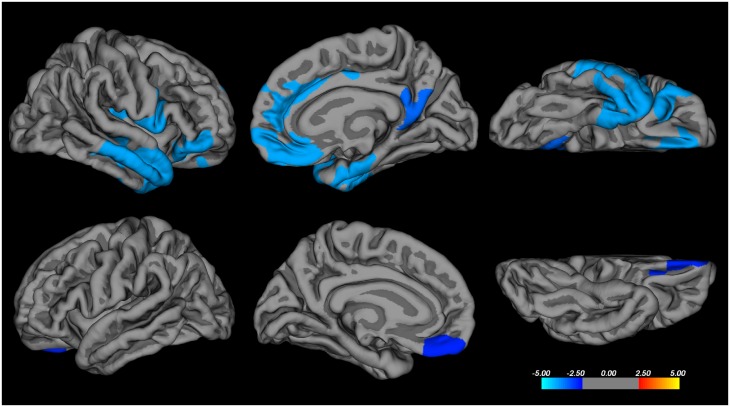
Cortical thinning in bvFTD patients. Results of the comparison of cortical thickness between patients with behavioural variant frontotemporal dementia and healthy controls. Results are from the Freesurfer analysis, results of the general linear model analysis were corrected for multiple comparisons at the cluster level using the Monte Carlo method for p-cluster at *p* < 0.01 (z-vertex 2.0). No nuisance variables were entered into the model. Coloured areas represent areas of significant differences, where warmer colours represent cortical thickening and cooler colours cortical thinning. Scale bar represents *p* on a logarithmic scale. The upper row is the right hemisphere, the lower row is left hemisphere, from lateral, medial and inferior views.

Compared with controls bvFTD patients showed widespread cortical thinning, bilaterally, with right hemispheric dominance (Fig 5). Thinning was significant (in the right hemisphere) in the medial prefrontal cortex (extending to the precuneus), the OFC (including the vmPFC), the inferior frontal gyrus/frontal operculum, and the insula. Regions of cortical thinning also included the temporal pole, extending to the middle and inferior temporal gyrus and parahippocampal gyrus. In the left hemisphere, cortical thinning was confined to the medial OFC/vmPFC. The PSP patients did not show any cortical thinning compared with controls, nor was there any statistically significant difference between patients with bvFTD and PSP. One patient with bvFTD had to be excluded from the analysis of cortical thickness because of incorrect parcellation.

### Group comparisons of white matter integrity

**Fig 6:**
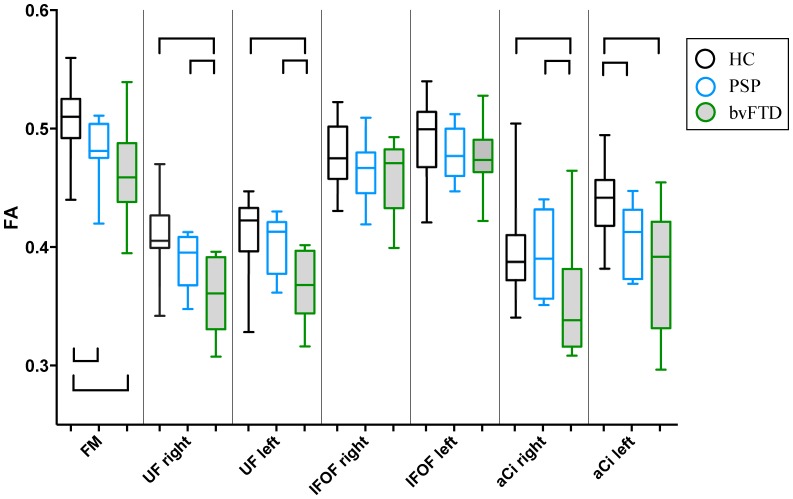
Boxplots of FA values of tracts studied. HC: healthy controls, PSP: progressive supranuclear palsy, bvFTD: behavioural variant frontotemporal dementia. FA: fractional anisotropy. FM: forceps minor, UF: uncinate fasciculus, IFOF: inferior frontooccipital fasciculus, aCi: anterior cingulum. Rh: right hemisphere and lh: left hemisphere. Boxes represent 25^th^ and 75^th^ percentile with median, whiskers minimum and maximum value. Staples represent statistically significant differences in between group pairs, at *p* < 0.05, uncorrected for multiple comparisons.

Boxplots of FA values of the tracts are shown in Fig 6. All tracts except the IFOF showed statistically significant differences across all three groups. Patients with bvFTD showed a robust and statistically significant lowering of FA in all tracts except the IFOF compared with healthy controls, whereas significant differences between PSP and controls were confined to the forceps minor and the left aCi. Comparisons between the two patient groups showed no difference in the forceps minor, IFOF or the left aCi, but lower FA in bvFTD in the right aCi and right and left UF compared with PSP. Values of mean diffusivity (MD) displayed a similar but reversed pattern (i.e. MD bvFTD>PSP>controls) (S1 Fig), the difference being that MD showed a statistical significant difference between bvFTD and controls also for the IFOF, there were no differences between PSP and controls, and bvFTD and PSP patients displayed statistically significant differences in the left aCi as well. Intra-raterreliability ranged from 0.93 to 0.99 and inter-raterreliability ranged from 0.89 to 0.98, in agreement with other centres using deterministic tractography with manually traced ROIs [51]. One patient with bvFTD had to be excluded from the DTI analysis because of movement during the scan.

## Discussion

The primary purpose of this study was to explore the clinico-anatomical correlates of disinhibition, adding the perspective of white matter to the more common grey matter imaging. The main finding of our study is that in the prefrontal syndrome disinhibition is related to the integrity of an axis between the right temporal-, insular- and orbitofrontal cortex, and their main interconnecting white matter structures, the right uncinate fasciculus and right cingulum. As such, our results have consequences for understanding the clinical anatomy of behavioural control. The secondary aim of the study, the comparison of the two syndromes bvFTD and PSP, will also be discussed in the following.

### Correlates of disinhibition in the present study

The second part of the Hayling test is specifically designed to tap inhibitory function [30] and thus to be a specific marker of disinhibition. In our study the total error score on the Hayling test correlated with white matter involvement of the right aCi, right UF, and forceps minor, but not the other tracts studied. This pattern is very similar to that in a combined neurodegenerative (bvFTD + AD) correlational study using VBM analysis of DTI and the Hayling test [11], with the difference that our findings were unilateral in the right hemisphere. The Hayling test has an embedded verbal component, but in the present study no association was seen with left hemispheric language areas. This is in agreement with a previous study of a bvFTD cohort [11], where verbal function did not influence results on the Hayling test. To quantify lack of inhibitory control outside the test setting, we chose a composite symptom score, FBI^12-22^. All FBI^12-22^ items include an aspect of inhibitory control, but the items and thus the composite score are likely to depend on other cerebral functions and regions as well. Eating disturbances, social cognition and aberrant motor behaviour are all symptoms of bvFTD and map into the OFC, medial PFC, insula and anterior temporal lobe [5, 52, 53]. However, the same tracts that correlated with the FBI^12-22^ symptom score correlated with the Hayling test (with the addition of the left UF), a considerably more “pure” disinhibition measure, which shows that our composite symptom score has a validity for the purpose of quantifying disinhibition in real life. The Hayling total error score correlated to reduced cortical thickness in the parahippocampal gyrus, OFC and insula, all in the right hemisphere, which, importantly, constitute a subset of the regions involved in our bvFTD patients. Taken together, our grey and white matter findings point to a right hemispheric medial temporal lobe (presumably including amygdala and hippocampus)-insular-orbitofrontal cortex network, areas which all are interconnected by the uncinate fasciculus and the cingulum [2].

### Consequences for the neuroanatomy of disinhibition

Measures of disinhibition in bvFTD, PSP and other neurodegenerative disorders tend to map into the OFC [5–8, 10, 11, 49, 54], which in the standard model of behavioural neurology is critical for behavioural inhibition [1, 3]. However, the studies are heterogeneous and several studies show a relationship to a considerably wider set of regions, mainly in the right hemisphere: the hippocampus/amygdala [9, 54], caudate/nucleus accumbens [9, 54], insula [8, 11], anterior cingulate cortex/medial prefrontal cortex [10, 54], inferior frontal gyrus [8, 10, 48, 49], and the temporal lobe [8, 10, 11, 54, 55] and other studies have shown both disinhibition and apathy correlating with the OFC [7, 8]. In healthy subjects the results are more consistent and inhibition (commonly assessed experimentally with motor inhibition) is related to function of the inferior frontal gyrus [56]. This has also emerged in FTD (see above) but shown to be secondary to OFC involvement [49]. In vivo white matter correlational studies are more recent, but point to the UF pathway for behavioural regulation both in acquired and developmental disorders [11, 14]. Comprehensively, we believe that the results of the present study taken together with the existing literature point to a right hemispherical medial temporal-insular-OFC axis for behavioural control, with the UF and Ci as the main structural linkage.

Interestingly, we did not find a correlation of disinhibition with cortical thickness of the vmPFC. Care should be taken when interpreting statistically non-significant findings as negative but the vmPFC did not correlate at a permissive, post-hoc *p* < 0.05 analysis, and was affected in our bvFTD cohort. This finding is in contrast to the majority of studies in bvFTD and PSP cited above, with the notable exception of the study by Zamboni et al [9]. Results from their study and our current agree with the models based on lesion and animal work [57–59], according to which the vmPFC is a critical hub for emotional decision making and/or evaluation of hedonistic experience, but not for inhibitory control per se, and extend these models to the context of neurodegenerative disease.

Our conclusion raises the broader question of why inhibitory control is anatomically “distributed” in such a manner. One explanation, using a locationalist view, is that not only behavioural but also neuropsychological measures of disinhibition are dependent on other functions in addition to inhibitory control which are localised in other cortical regions, communicate by the white matter pathways, and will “contaminate” the neuroimaging results. As an example, social cognition is coupled to the anterior temporal lobe [60], medial PFC is coupled to mentalisation and decision making [61], the amygdala to emotional processing [62] and the medial temporal lobe to contextual information [63]. An alternative explanation is an associative network model, where the inhibitory function itself relies on a distributed network with several nodes, i.e. parts of the network that each contribute to but are not individually critical for its function [13, 64]. We believe that the results of the present study, where behavioural and neuropsychological measures of disinhibition correlate with distributed right hemispheric cortical areas and their interconnections is more in line with the latter model. This we believe could explain some of the divergences among previous studies. Such a model however does question the concept of a “prefrontal” syndrome as currently conceptualized [65].

### Grey and white matter affliction in bvFTD vs. PSP

The regions affected by cortical atrophy among the bvFTD patients in our study (medial prefrontal, orbitofrontal, inferior frontal, insular and temporal cortex) are consistent with the expected pattern in bvFTD [23], although our patients showed a right-sided dominance. The involvement of major associative and commissural tracts leading to/from these areas [2, 14, 66], i.e. the aCi, UF and forceps minor, is also in accordance with previous studies on bvFTD in which these are the most consistently affected tracts [50, 67–69].

In the PSP patients we did not find any statistically significant cortical thinning compared with controls, which was contrary to expectations since the PSP patients had prefrontal symptomatology (“Richardson´s syndrome”). In PSP these symptoms are related to both subcortical and prefrontal cortical affliction [6, 22, 70, 71]. It is possible that measuring cortical thinning is a less sensitive method than those used in the other studies, or an issue of power. On the other hand, the pattern of tract involvement (forceps minor and left aCi) is in accordance with previous DTI tractographical studies of PSP [72–74].

The symptomatology of our patients confirmed that it is the FBI “positive” symptoms (FBI^12-22^) that, from a behavioural perspective, best separates PSP and bvFTD, while the “negative” symptom (FBI^1-10^) burden is more similar [15, 17, 26]. Adding to the topographical overlap of the conditions, with a variable extent of prefrontal and subcortical atrophy [6, 20–23, 70, 71], we believe this supports the use of a combined disease cohort of bvFTD and PSP patients when exploring the underlying neuroanatomy of disinhibition. Lagarde et al. [71] have made the only previous direct grey matter atrophy comparison between bvFTD and PSP and did not find any differences when using their a priori threshold, as in this study. However, using a post-hoc, more permissive threshold they showed greater atrophy in dorsolateral, medial and orbital prefrontal cortex in bvFTD compared with PSP. Similarly, when we used a more permissive multiple comparison cut-off (*p* < 0.05) we did indeed find a cortical thinning in bvFTD compared with PSP, in the OFC (including the vmPFC) and anterior temporal lobe (S2 Fig). To our knowledge our study is the first direct DTI comparison between bvFTD and PSP using DTI for comparing white matter integrity in the two disorders. Comparison between the two disease groups showed lower FA in bvFTD in the right aCi and the UF bilaterally compared with PSP. This itself is an indication that the phenotype distinguishing bvFTD from PSP (that is the “positive” FBI^12-22^ symptomatology) is related to the involvement of these tracts.

### Methodological discussion

From a clinico-anatomic-correlation standpoint, it is important to note that all tracts we studied, although they constitute anatomically discrete fibre systems, are composed of subcomponents which likely have different functions and possibly different involvement in the diseases studied. For example, the least affected tract in the bvFTD group, the IFOF, is composed of several subcomponents [75] that are not separated in the present study. Another limitation of the DTI method employed is that fibres that cross or”kiss” other fibres will be excluded which limits the extents of the tracts in a non-anatomical fashion [12]. Also, the current DTI resolution means that DTI parameters of tracts will be contaminated from adjoining or intermingling tracts. An example of this problem is in the orbitofrontal terminations of the UF and IFOF, which will be intermingled [2]. Future studies with methods that enable more detailed tract parcellations may overcome some of these problems [12]. The imaging methods chosen for the current study do not visualize the cortico-striatal-thalamic loop. Considering its role in inhibitory control, and (as noted previously) its affliction in bvFTD and PSP, both grey and white matter imaging of this system would constitute an important improvement.

A broader methodological aspect of this study lies in the use of the clinico-anatomical-correlation method in neurodegenerative diseases. Neurodegenerative diseases, through the regional selectivity of their respective pathological processes, probably reflect entire large-scale functional-structural networks [76]. This could pose a limitation, making it difficult to disentangle separated functions in an afflicted network even with a hodological approach [77].

## Conclusion

In conclusion, we show that disinhibition is related to the integrity of a right hemispheric medial temporal-insular-orbitofrontal network, interconnected by the right uncinate fasciculus and right cingulum. These results support a frontotemporal network model of disinhibition, and question the concept of a purely prefrontal syndrome as currently formulated. Also, our study questions the role of atrophy of the ventromedial prefrontal cortex as the major cause for loss of inhibitory control in neurodegenerative disease.

## Supporting Information

S1 Fig Boxplots of MD values of tracts studied. HC: healthy controls, PSP: progressive supranuclear palsy, bvFTD: behavioural variant frontotemporal dementia. MD: mean diffusivity. FM: forceps minor, UF: uncinate fasciculus, IFOF: inferior frontooccipital fasciculus, aCi: anterior cingulum. Rh: right hemisphere and lh: left hemisphere. Boxes represent 25^th^ and 75^th^ percentiles with median, whiskers minimum and maximum value. Staples represent statistically significant differences between paired groups, at *p* < 0.05, uncorrected for multiple comparisons.
(EPS)

S2 Fig Comparison of cortical thickness between patients with bvFTD and PSP. Results of the post-hoc comparison of cortical thickness between patients with behavioural variant frontotemporal dementia (bvFTD) and progressive supranuclear palsy (PSP) using Freesurfer. Results of the general linear model analysis were corrected for multiple comparisons at the cluster level using the Monte Carlo method for p-cluster at *p* < 0.05 (z-vertex 2.0). No nuisance variables were entered into the model. Coloured areas represent areas of significant differences, where warmer colours represent cortical thickening and cooler colours cortical thinning. Scale bar represents p on a logarithmic scale. Only the right hemisphere is shown, from lateral, medial and inferior views. No regions with significant correlation with cortical thickness were detected in the left hemisphere.
(EPS)
